# The metabolic layer of cognition: integrating metabolomics, breathomics, and systems neuroscience

**DOI:** 10.3389/fnins.2026.1842643

**Published:** 2026-06-24

**Authors:** Bianca Bonato, Umberto Castiello

**Affiliations:** Department of General Psychology, University of Padova, Padua, Italy

**Keywords:** breathomics, cognitive neuroscience, mass spectrometry, metabolomics, neuroenergetics, volatile organic compounds

## Abstract

Cognitive neuroscience has made substantial progress in mapping neural activity underlying perception, memory, and decision-making. However, widely used methods such as functional magnetic resonance imaging and electrophysiology primarily measure indirect physiological correlates of neuronal activity and provide limited access to the biochemical processes that support neural signaling. In this review, we propose that metabolism might constitutes a critical intermediate layer linking neural activity and behavior. Drawing on advances in metabolomics and breathomics, we examine how mass spectrometry-based analytical techniques enable sensitive detection of metabolites, neurotransmitters, lipids, and volatile organic compounds that could reflect metabolic processes associated with neuronal signaling and cognitive states. We synthesize emerging research at the intersection of neuroenergetics, systems neuroscience, and metabolic profiling, highlighting how these approaches can complement established neuroimaging and electrophysiological methods. In particular, we discuss the potential of volatile organic compounds in exhaled breath as non-invasive indicators of systemic metabolic responses accompanying cognitive processes. At the same time, we address key conceptual and methodological challenges in interpreting peripheral metabolic signals in relation to brain activity, including the influence of systemic physiology, microbiome metabolism, and environmental factors. Finally, we outline future directions for integrating metabolomic and breathomic measurements with neural and behavioral data in multimodal experimental frameworks. Incorporating metabolic dynamics into systems-level models may provide a new perspective on how cognition emerges from interactions between brain activity and whole-body physiology.

## 1 Introduction

Cognitive neuroscience seeks to explain how neural processes give rise to perception, memory, emotion, and decision-making. Over the past several decades, neuroimaging and electrophysiological techniques have enabled increasingly precise mapping of the neural correlates of cognitive functions (Logothetis, 2008; Raichle, 2015). Despite these advances, dominant methodological approaches primarily capture indirect physiological signatures of neural activity-such as blood oxygenation or electrical fields, while providing limited access to the molecular and biochemical processes that sustain neuronal signaling (Raichle and Mintun, 2006). As a result, current models of cognition remain largely centered on neural dynamics, with comparatively less attention devoted to energetic and biochemical processes that enable and constrain them.

A growing body of research in neuroenergetics challenges this imbalance by demonstrating that neural computation is tightly coupled to cellular metabolism and energetic resource allocation (Attwell and Laughlin, 2001; Harris et al., 2012; Magistretti and Allaman, 2015). Synaptic transmission, action potential generation, and large-scale network activity all impose substantial energetic demands, supported by coordinated interactions between neurons, astrocytes, mitochondria, and vascular systems. These findings suggest that cognitive processes cannot be fully understood without considering the metabolic systems that sustain neural activity.

**Figure 1:**
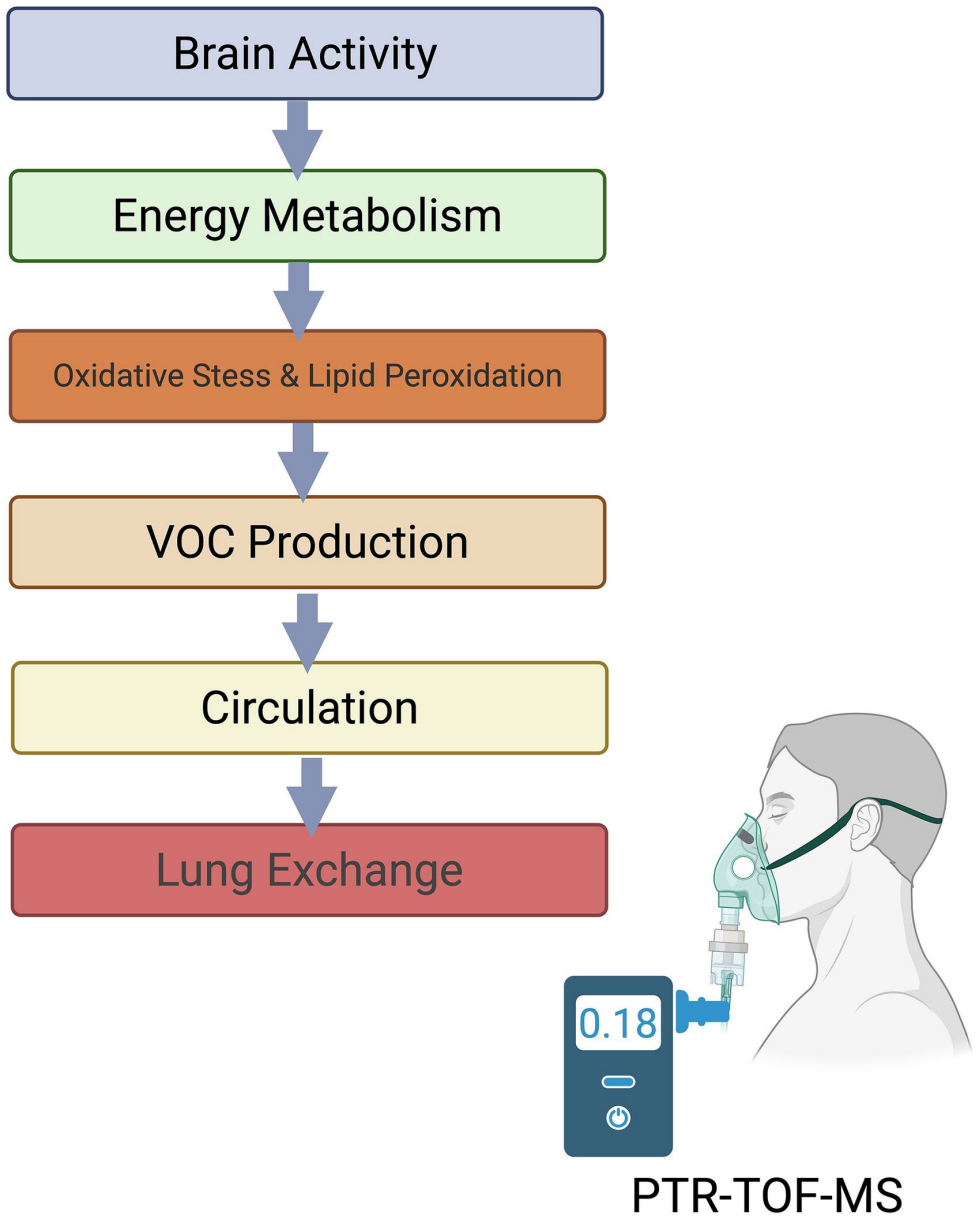
From neural metabolism to volatile compounds in breath. Neural activity increases cellular energy demand and metabolic turnover, generating biochemical by-products, including volatile organic compounds (VOCs). Some VOCs enter the bloodstream and are subsequently released through pulmonary gas exchange, providing a non-invasive window into brain-related metabolic processes.

Building on these insights, we propose that metabolism might constitutes an intermediate level of analysis linking neural activity and behavior. We introduce the concept of a metabolic layer of cognition, which conceptualizes cognitive processes as emerging from interactions between neural computation, cellular energetics, and whole-body metabolic regulation. Within this framework, metabolic dynamics are not merely downstream consequences of neural activity but integral components of a distributed physiological system that both constrains and reflects cognitive function. This conceptual relationship between neural activity, metabolic processes, and systemic physiological signals is illustrated in Figure 1.

Advances in mass spectrometry (MS) have made it possible to access this metabolic layer with unprecedented sensitivity and breadth. MS-based metabolomics enables the detection and quantification of metabolites, neurotransmitters, lipids, and peptides across a wide range of biological samples (Patti et al., 2012; Aebersold and Mann, 2016). When combined with chromatographic techniques such as gas chromatography (GC) or liquid chromatography (LC), these approaches allow large-scale characterization of biochemical pathways associated with cellular and systemic metabolism (Dettmer et al., 2007). Importantly, these methods provide a molecular perspective that complements traditional neuroimaging and electrophysiological measurements by capturing the biochemical substrates of neural activity.

In parallel, increasing attention has been directed toward volatile organic compounds (VOCs), a diverse class of small carbon-based molecules produced by endogenous metabolic processes and detectable in exhaled breath and other biofluids (Amann and Smith, 2013; de Lacy Costello et al., 2014). Breathomics, the analysis of VOCs in exhaled breath, offers a particularly promising non-invasive approach for monitoring metabolic activity in real time. Technologies such as proton-transfer-reaction time-of-flight mass spectrometry (PTR-TOF-MS) enable highly sensitive detection of trace compounds, opening new possibilities for studying physiological responses during cognitive tasks (Jordan et al., 2009).

From the perspective developed in this review, metabolites and VOCs can be understood as systemic molecular signals reflecting metabolic processes associated with neural activity and cognitive demand. Neural computation generates energetic requirements that engage cellular metabolism within the brain while simultaneously triggering organism-level responses involving vascular regulation, peripheral organs, and circulating metabolites. Metabolomic and breathomic measurements provide experimental access to these processes, offering a complementary window into the physiological mechanisms underlying cognition.

At the same time, interpreting metabolic signals poses substantial conceptual and methodological challenges. Many metabolites and VOCs originate from multiple tissues and biochemical pathways, making it difficult to attribute peripheral measurements specifically to neural processes. In addition, metabolic profiles are influenced by factors such as diet, microbiome composition, circadian rhythms, and environmental exposure (Amann et al., 2014; Beauchamp and Pleil, 2013). These considerations underscore the need for cautious interpretation and for multimodal experimental designs that integrate metabolic measurements with neural and behavioral data.

The aim of this review is to synthesize research at the intersection of metabolomics, breathomics, and systems neuroscience in order to develop a conceptual framework for studying cognition as a metabolically embedded process. We argue that incorporating metabolic dynamics into cognitive neuroscience can extend existing models by linking neural activity with biochemical and physiological processes distributed across the brain and body. In the following sections, we review analytical techniques for measuring metabolic processes, examine evidence linking metabolic dynamics to cognitive function and brain disorders, and outline experimental strategies for integrating metabolic and neural measurements within unified systems-level models of cognition.

## 2 Relation to existing frameworks

The metabolic framework of cognition proposed here builds on and extends several established approaches that emphasize the interplay between brain function and physiological regulation. In particular, it is closely related to research in neuroenergetics, embodied cognition, and models of brain–body interaction, while introducing a distinct focus on measurable metabolic signals as an intermediate level of analysis.

Neuroenergetic accounts have long demonstrated that neural activity is tightly coupled to energy metabolism, highlighting the role of glucose utilization, mitochondrial function, and astrocyte–neuron interactions in supporting synaptic transmission (Attwell and Laughlin, 2001; Harris et al., 2012; Magistretti and Allaman, 2015). The present framework builds on these insights but extends them in two key ways. First, it emphasizes that metabolic processes are not only constraints on neural computation but also sources of measurable physiological signals accessible through metabolomic and breathomic approaches. Second, it expands the focus from cellular and local brain metabolism to distributed metabolic dynamics spanning brain and peripheral systems.

The framework also shares conceptual ground with embodied and systems-based perspectives, which view cognition as emerging from interactions between neural processes and bodily states. However, while these approaches often emphasize functional or dynamical coupling between brain and body, they typically lack a direct account of the biochemical substrates underlying these interactions. By contrast, the metabolic framework explicitly incorporates molecular and biochemical measurements, providing a potential bridge between systems-level theories and experimentally accessible physiological signals.

Finally, the proposed framework aligns with broader models of brain–body integration, including those emphasizing allostasis and physiological regulation, in which cognitive processes are shaped by the need to maintain internal stability in changing environments. Within this context, metabolic signals can be understood as reflecting ongoing regulatory processes that coordinate neural activity with organism-level energetic demands.

Taken together, the metabolic framework does not replace existing theories but complements them by introducing a molecular and biochemical perspective on brain–body interactions. By positioning metabolism as an intermediate and measurable layer linking neural dynamics with behavior, this approach provides a conceptual and methodological extension of current models of cognition.

## 3 Mass spectrometry as a molecular tool for neuroscience

Mass spectrometry (MS) provides a powerful approach for accessing the molecular substrates of physiological processes underlying cognition. Rather than directly measuring neural activity, MS-based techniques enable the detection of metabolites, neurotransmitters, lipids, and volatile compounds that reflect the biochemical dynamics associated with neural computation and systemic metabolic regulation.

In neuroscience research, MS is particularly valuable because it allows the characterization of molecular profiles across a wide range of biological samples. This section focuses on analytical platforms most relevant for studying metabolic processes associated with neural activity and behavior, with an emphasis on their potential to complement established neuroimaging and electrophysiological methods.

**Figure 2:**
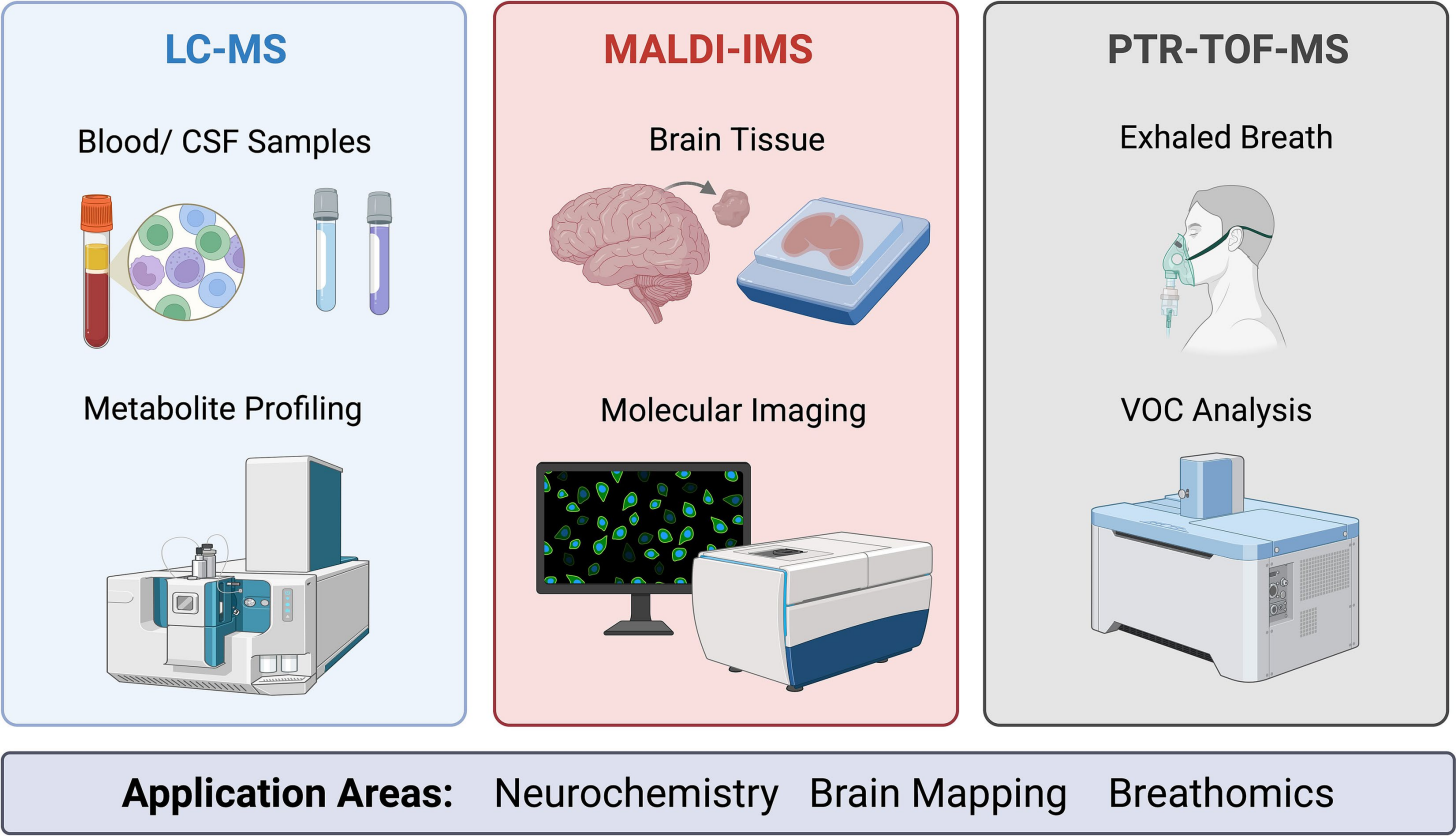
Mass spectrometry approaches for studying brain metabolism. Complementary mass spectrometry approaches enable metabolic measurements across different biological scales. LC–MS provides metabolite profiling in biofluids, MALDI imaging mass spectrometry (MALDI-IMS) reveals spatial molecular distributions in brain tissue, and PTR-TOF-MS enables real-time detection of VOCs in exhaled breath.

This review emphasizes MS-based approaches used in metabolomics and breathomics (Figure 2; Table 1), which provide access to metabolic signals distributed across brain and body systems. A central challenge in this context is determining whether detected metabolites reflect neural processes within the central nervous system or broader physiological activity. Evidence from studies of brain tissue and cerebrospinal fluid indicates that metabolic pathways associated with oxidative phosphorylation, lipid turnover, and neurotransmitter synthesis are closely coupled to neuronal signaling (Hyder et al., 2013; Dienel, 2019).

**Table 1:** Mass spectrometry approaches used in cognitive neuroscience research.

Mass spectrometry platform	Typical biological samples	Molecular classes detected	Representative applications in neuroscience	Key methodological considerations	References
LC–MS metabolomics	Blood, plasma, cerebrospinal fluid (CSF), brain tissue	Polar metabolites, lipids, neurotransmitter precursors	Metabolic pathway profiling, biomarker discovery in neurodegenerative and psychiatric disorders	Peripheral metabolites may only indirectly reflect brain-specific metabolic activity	Dettmer et al. (2007), Patti et al. (2012), Aebersold and Mann (2016)
GC–MS	Breath, plasma, urine	Volatile organic compounds (VOCs), small metabolites	Breathomics studies, oxidative stress markers, systemic metabolic signatures	VOCs can be influenced by environmental exposure, diet, and microbiome activity	Dettmer et al. (2007), de Lacy Costello et al. (2014), Amann et al. (2014)
PTR-TOF-MS	Exhaled breath	Trace VOCs measured in real time	Real-time monitoring of metabolic responses during cognitive tasks or stress	Limited molecular identification without complementary analytical techniques	Jordan et al. (2009), Amann and Smith (2013), Avesani et al. (2026)
MALDI imaging mass spectrometry	Brain tissue sections	Peptides, lipids, metabolites	Spatial mapping of molecular distributions across brain regions	Typically requires post-mortem or invasive sampling	Aebersold and Mann (2016), Graham et al. (2015)
LC–MS combined with microdialysis	Extracellular brain fluid	Neurotransmitters and metabolic intermediates	Monitoring neurochemical dynamics during behavior or pharmacological manipulation	Highly invasive and mainly applied in animal models	Kennedy et al. (2002), Ungerstedt (1991)

Overview of commonly used mass spectrometry techniques, biological samples, molecular classes detected, typical neuroscience applications, and methodological limitations.

Electrospray ionization (ESI) is widely used in biological research because it enables the analysis of polar and high-molecular-weight biomolecules in solution (Banerjee and Mazumdar, 2012). It is particularly well suited for detecting metabolites, peptides, and signaling molecules relevant to neuroscience. In the context of cognitive neuroscience, ESI-based approaches enable the detection of biochemical changes associated with neural activity and organism-level metabolic responses.

Matrix-assisted laser desorption ionization (MALDI) is another soft ionization technique commonly used for analyzing large biomolecules such as proteins and peptides. In neuroscience, MALDI is particularly valuable for imaging mass spectrometry approaches that map molecular distributions directly within brain tissue (Aebersold and Mann, 2016). This capability provides spatially resolved information on biochemical organization, offering a potential link between regional brain activity and underlying metabolic processes.

### 3.1 Time-of-flight mass spectrometry

Time-of-flight (TOF) mass spectrometry is widely used in metabolomics and volatile organic compound (VOC) analysis due to its high acquisition speed and broad mass range (Cotter, 1999; Avesani et al., 2025). TOF-based instruments enable rapid acquisition of full mass spectra, making them particularly suitable for detecting dynamic changes in metabolic activity and capturing transient physiological responses.

TOF analyzers provide high mass accuracy and resolution, which are essential for identifying metabolites in complex biological samples. Quadrupole-time-of-flight (QTOF) instruments further extend these capabilities by combining targeted and untargeted analysis within a single platform (Hopfgartner et al., 2004), making them widely used in metabolomics and biomarker discovery.

An important application of TOF technology in this context is proton-transfer-reaction mass spectrometry (PTR-TOF-MS), which enables real-time detection of volatile organic compounds at extremely low concentrations (Jordan et al., 2009; Avesani et al., 2026). The recently developed adduct ionization mechanism (AIM) reactor further expands the detectable range of compounds, potentially improving sensitivity in breath analysis applications (Avesani et al., 2026). These developments are particularly relevant for cognitive neuroscience, as they enable continuous, non-invasive monitoring of metabolic responses during task performance, providing a potential link between cognitive processes and real-time physiological dynamics.

Taken together, these analytical approaches do not directly measure neural activity but provide complementary access to the metabolic processes that support and accompany cognitive function. As such, they form a methodological foundation for investigating the metabolic layer of cognition proposed in this review.

## 4 Metabolomics and breathomics: systemic metabolic signals

Volatile organic compounds (VOCs) are small carbon-based molecules that readily evaporate at room temperature due to their high vapor pressure and relatively low molecular weight. They encompass a broad range of chemical classes, including alcohols, aldehydes, ketones, hydrocarbons, esters, and sulfur-containing compounds (Dudareva et al., 2014). VOCs are ubiquitous in both environmental and biological systems; however, in humans they are of particular interest because many are thought to arise, at least in part, as by-products of endogenous metabolic processes and may therefore reflect physiological or pathological states (Amann and Smith, 2013; de Lacy Costello et al., 2014). Despite the promise of VOC analysis, several limitations must be considered when interpreting breathomic data. Many volatile compounds originate from multiple tissues and metabolic pathways, making it difficult to attribute specific signals directly to neural processes. Environmental exposure, diet, microbiome composition, and circadian rhythms can also influence VOC profiles. Furthermore, substantial inter-individual variability represents an additional challenge for the interpretation of peripheral metabolomic and breath VOC measurements. Factors such as age, sex, genetic background, lifestyle, medication use, health status, and baseline metabolic phenotype can significantly shape VOC composition, potentially limiting the generalizability of findings across populations (Amann et al., 2014). VOC profiles may also exhibit temporal instability within the same individual, varying across days or even within shorter time windows due to fluctuations in physiological state, stress, sleep, physical activity, or recent food intake (Basanta et al., 2012; King et al., 2010). In addition, methodological differences in breath collection procedures, sensor technologies, analytical pipelines, and statistical preprocessing can substantially affect reproducibility across studies (Lourenço and Turner, 2014). These limitations highlight the need for standardized acquisition protocols, longitudinal designs, larger validation cohorts, and replication across independent datasets before breathomic biomarkers can be reliably translated into robust indicators of cognitive or neural states (Hanna et al., 2019).

Consequently, rigorous experimental design and multimodal validation with neural or physiological measures are essential when using breath VOCs as indicators of cognitive states (Beauchamp and Pleil, 2013; Amann et al., 2014).

**Figure 3:**
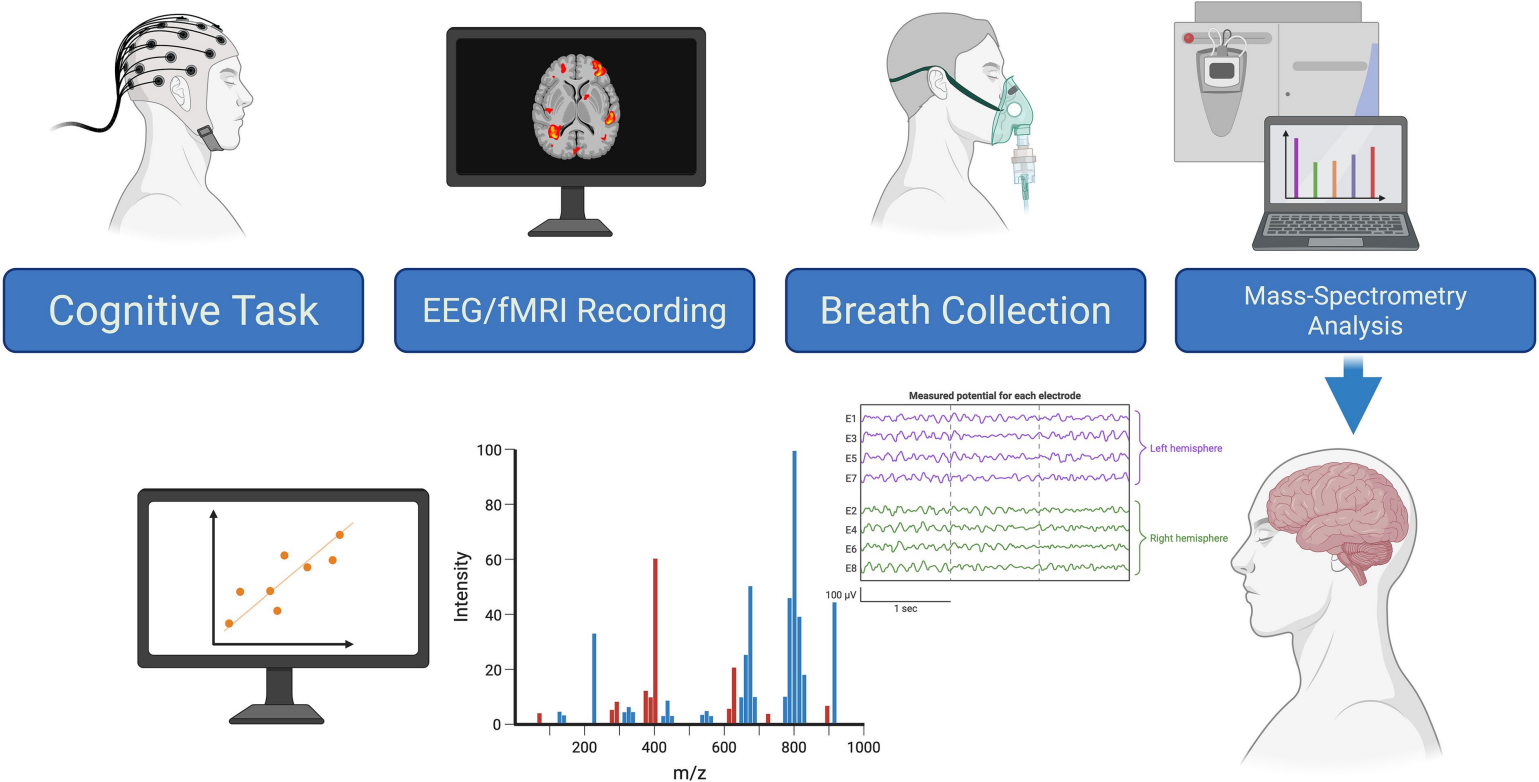
Multimodal integration of neural and metabolic measurements. Simultaneous acquisition of neural signals (e.g., EEG or fMRI) and breath metabolomic data during cognitive tasks allows investigation of how metabolic changes relate to brain activity and behavioral performance.

Importantly, VOC measurements should not be interpreted as direct readouts of neural activity. Instead, breathomic signals are better understood as indicators of systemic metabolic responses associated with neural activity and cognitive demand. Cognitive processes increase energetic requirements within neural circuits, which in turn engage broader metabolic and physiological regulatory systems. Breath VOCs may therefore provide indirect but informative markers of these systemic metabolic responses accompanying neural computation (Figure 3).

In humans, VOCs originate from multiple biochemical pathways. They are produced through biochemical pathways involving oxidative stress, lipid peroxidation, microbial activity, and enzymatic reactions (Amann and Smith, 2013). For example, oxidative degradation of polyunsaturated fatty acids in cell membranes generates alkanes and aldehydes, which can enter the bloodstream and ultimately be exhaled (Phillips et al., 1999). In addition, microbial metabolism in the gut and oral cavity contributes a range of volatile metabolites through fermentation and other microbial pathways (de Lacy Costello et al., 2014). Once formed, these compounds can circulate systemically and reach the lungs, where they diffuse across the alveolar-capillary membrane and are released into exhaled breath.

## 5 Metabolic signatures of cognition and brain disorders

Metabolomic measurements obtained through mass spectrometry (MS) provide important insights into biochemical processes associated with neural activity (Nicholson et al., 1999). Because neuronal signaling is energetically demanding, metabolic signatures measured by MS may help characterize systemic biochemical states that accompany cognitive processes and their underlying neuroenergetic requirements (Patti et al., 2012; Raichle, 2015). Neural computation requires substantial energy expenditure, and cognitive demands can influence glucose utilization, mitochondrial activity, and oxidative balance. Metabolomic approaches therefore provide a complementary perspective on physiological processes associated with cognition alongside electrophysiological and neuroimaging methods.

Several metabolic pathways may plausibly contribute to associations between cognitive activity and measurable metabolic profiles. These include changes in glucose utilization during cognitive demand, mitochondrial oxidative phosphorylation supporting synaptic transmission, and oxidative stress pathways associated with lipid peroxidation (Dienel, 2019; Bélanger et al., 2011). Processes related to glutamate metabolism and glutamate-glutamine cycling are also central to brain energetics and neurotransmission; however, peripheral measurements of glutamate-related metabolites should not be interpreted as direct markers of central glutamatergic neurotransmission because central and peripheral glutamate pools are tightly regulated and largely separated by the blood–brain barrier (Hawkins, 2009; Hawkins and Viña, 2016). Indeed, glutamate transport across the blood–brain barrier is highly restricted under physiological conditions, limiting direct correspondence between circulating and brain glutamate concentrations (Hawkins and Viña, 2016). Accordingly, metabolomic measurements may provide only indirect indicators of broader energetic, neuroendocrine, immune, or systemic biochemical processes associated with neural computation during cognitively demanding tasks.

An additional factor that may substantially influence the relationship between metabolism and cognition is sleep and circadian regulation. Sleep plays a fundamental role in cerebral energy homeostasis, mitochondrial respiration, glymphatic metabolite clearance, redox balance, synaptic plasticity, and neuroendocrine regulation, all of which are closely linked to cognitive performance and neural integrity. Experimental and clinical studies have demonstrated that sleep disruption and circadian misalignment can profoundly alter systemic metabolism, inflammatory signaling, oxidative stress pathways, and peripheral metabolomic profiles. Conditions such as chronic sleep restriction and obstructive sleep apnea are associated with intermittent hypoxia, mitochondrial dysfunction, immunometabolic alterations, increased lipid peroxidation, and impaired glucose regulation, which may contribute to cognitive dysfunction and neurodegenerative vulnerability (Mohit et al., 2023a,b; Pateriya et al., 2022).

Emerging metabolomic evidence further indicates that circadian rhythms strongly influence the temporal organization of circulating metabolites, including amino acid metabolism, lipid metabolism, hormonal regulation, and oxidative pathways, thereby introducing substantial temporal variability into peripheral metabolomic measurements (Mohit et al., 2022; Mohit et al., 2023a,b). Circadian oscillations may therefore represent an important source of biological variability in both metabolomic and breathomic studies, particularly when sample collection timing is not rigorously standardized. Moreover, sleep-dependent metabolic dysregulation has been increasingly linked to cognitive impairment, neuroinflammation, and altered energetic coupling within frontoparietal and hippocampal networks involved in attention, executive function, and memory processes (Mohit et al., 2023a,b; Mohit et al., 2025). Collectively, these findings highlight the importance of integrating sleep physiology and circadian biology into metabolomic investigations of cognition and brain disorders, particularly in longitudinal and translational research frameworks.

Although such measurements cannot be interpreted as direct readouts of specific neurotransmitter activity in the brain, they may nevertheless contribute to the study of core cognitive processes by characterizing systemic physiological states associated with cognition. Functions such as attention, working memory, and cognitive control rely heavily on metabolically demanding frontoparietal and prefrontal networks. Neuroenergetic studies have shown that increased cognitive demand is accompanied by changes in glucose utilization, oxidative metabolism, and neuromodulatory signaling (Raichle, 2015). For example, prefrontal cortical activity underlying working memory and executive control is associated with substantial metabolic regulation (Arnsten, 2009). Similarly, theoretical models of cognitive control and decision making emphasize the role of distributed neural systems that dynamically allocate metabolic resources to support goal-directed behavior (Miller and Cohen, 2001; Shenhav et al., 2013). Metabolomic approaches may therefore provide a complementary window into the biochemical mechanisms supporting attention, decision making, and executive control.

Beyond general cognitive processes, metabolomic studies have also identified metabolic alterations associated with learning and memory. For example, metabolomic profiling of the hippocampus has revealed learning-related changes in metabolites associated with antioxidant pathways and cellular metabolism that may contribute to memory formation (Bessières et al., 2021). Experimental studies further suggest that energetic regulation of glutamate cycling, mitochondrial respiration, and antioxidant pathways is involved in synaptic plasticity and long-term memory formation (Bessières et al., 2021; Harris et al., 2012). However, many of these metabolic processes, particularly those related to glucose utilization and cellular energy metabolism, participate broadly in physiological functions and are therefore not uniquely specific to individual cognitive states or disease entities. Accordingly, metabolomic profiling may provide useful molecular-level information about biochemical processes associated with neuronal plasticity and learning, while interpretations regarding disease- or process-specific mechanisms should be made cautiously.

In addition to studying normal brain function, MS-based metabolomics has a growing role in biomarker discovery for neurological and psychiatric disorders (Table 2). Metabolomic studies have identified metabolic alterations associated with conditions such as Alzheimer’s disease, Parkinson’s disease, depression, schizophrenia and attention-deficit/hyperactivity disorder (ADHD) (Trushina and Mielke, 2014; Michell et al., 2008; Kaddurah-Daouk et al., 2013; Yadav et al., 2025). For instance, metabolomic analyses have revealed altered lipid metabolism in Alzheimer’s disease as well as disruptions in dopamine metabolism, mitochondrial pathways, and oxidative stress in Parkinson’s disease (Trushina et al., 2013). Recent evidence has also suggested altered amino acid metabolism, oxidative stress pathways, and energetic dysregulation in children and adolescents with ADHD, supporting the possibility that peripheral metabolomic signatures may contribute to the characterization of neurodevelopmental and psychiatric conditions (Yadav et al., 2025). These findings demonstrate that metabolomic profiling can reveal disease-specific metabolic signatures and contribute to understanding the biochemical mechanisms underlying neurological disorders.

**Table 2:** Metabolic signatures associated with cognitive processes and neurological disorders.

Cognitive or clinical context	Biological sample	Representative metabolic alterations	Analytical approach	Example references
Alzheimer’s disease	Plasma/CSF	Alterations in lipid metabolism and mitochondrial energy pathways	LC–MS metabolomics	Trushina and Mielke (2014)
Parkinson’s disease	Sebum/plasma	Changes in lipid metabolites and oxidative stress markers	GC–MS or LC–MS metabolomics	Trivedi et al. (2019), Graham et al. (2018)
Major depressive disorder	Plasma	Altered amino-acid metabolism and lipid metabolic pathways	LC–MS metabolomics	Liu et al. (2021), Kaddurah-Daouk et al. (2013)
Learning and memory processes	Hippocampal tissue	Changes in antioxidant pathways and mitochondrial metabolism	LC–MS metabolomics	Bessières et al. (2021)
Acute stress responses	Exhaled breath	Increased isoprene, acetone, and aldehydes reflecting metabolic stress	PTR-TOF-MS breathomics	King et al. (2010), Amann and Smith (2013)
High cognitive load	Exhaled breath	Task-related shifts in volatile organic compound profiles	Breathomics (GC–MS or PTR-MS)	de Lacy Costello et al. (2014)

Summary of representative metabolomic and breathomic findings linking metabolic alterations to cognitive processes and neuropsychiatric conditions.

A complementary approach involves the analysis of volatile organic compounds (VOCs) in exhaled breath. Breath VOC measurements using proton-transfer-reaction time-of-flight mass spectrometry (PTR-TOF-MS) allow rapid and repeated sampling without invasive procedures (Jordan et al., 2009). Because VOCs can be detected in exhaled breath, breathomics provides a non-invasive window into metabolic processes occurring throughout the body. Breath analysis has been investigated for the diagnosis of lung cancer, infectious diseases, and metabolic disorders (Phillips et al., 1999). In addition, VOCs may reflect biochemical processes associated with neural activity, neuroinflammation, and oxidative stress. For example, increased levels of certain aldehydes and hydrocarbons in breath have been associated with neurodegenerative diseases such as Parkinson’s disease and Alzheimer’s disease (Trivedi et al., 2019; Wang et al., 2014).

Breathomics studies have also explored the relationship between VOC profiles and psychological states. Experimental research suggests that metabolic signatures in breath may change in response to stress, cognitive load, and emotional stimuli (de Lacy Costello et al., 2014). Controlled laboratory studies have shown that acute psychological stress can alter breath concentrations of compounds such as isoprene, acetone, and aldehydes, reflecting stress-related changes in lipid peroxidation and energy metabolism (King et al., 2010; Amann and Smith, 2013). Similarly, increases in certain VOCs have been observed during tasks involving high cognitive load, suggesting that metabolic activity associated with increased neuronal demand may be reflected in breath chemistry (de Lacy Costello et al., 2014). Emotional states have also been associated with distinct VOC patterns; for instance, exposure to fear-inducing stimuli can modify the composition of human body odor and breath volatiles, potentially reflecting stress-induced metabolic and hormonal responses (de Groot et al., 2012).

Although these findings suggest potential links between metabolic signatures and cognitive states, direct evidence connecting breath VOC dynamics to specific neural computations remains limited. Most existing studies measure global physiological responses to stress or cognitive load rather than identifying metabolite patterns uniquely associated with cognitive operations. Future research will therefore require carefully controlled experiments that combine breathomic measurements with simultaneous neuroimaging, electrophysiology, or behavioral paradigms. Such multimodal approaches will be necessary to determine whether metabolic signatures reliably track cognitive processes rather than broader physiological arousal or systemic energetic regulation.

These findings are particularly relevant for forensic psychology, where objective physiological indicators of psychological states are often sought. VOC-based breath analysis could potentially contribute to the assessment of stress, deception, or heightened cognitive effort during investigative interviews or interrogations, as deceptive responses are known to increase cognitive load and physiological arousal (Vrij et al., 2017). In addition, breathomic markers of acute stress or anxiety could provide complementary physiological information during forensic evaluations or in studies examining emotional responses to threatening or crime-related stimuli (Lucchi et al., 2024). Although such applications remain largely exploratory, these findings raise the possibility that VOC measurements could serve as physiological indicators of cognitive and emotional states relevant to forensic contexts.

## 6 Integrating metabolomics with systems neuroscience

Because MS measures molecular composition rather than neural activity itself, it is particularly informative when integrated with established methods in cognitive neuroscience. Multimodal approaches combining MS with neuroimaging, electrophysiology, and behavioral measures can link molecular processes with neural dynamics and cognitive performance, thereby providing a more comprehensive understanding of the biological foundations of cognition. A central challenge is establishing causal relationships between metabolic signatures and neural computation. Combining time-resolved metabolomics with electrophysiology, neuroimaging, and behavioral analysis may enable systems-level models linking molecular metabolism with large-scale brain dynamics (Raichle, 2015; Dienel, 2019). Metabolic markers frequently reflect systemic physiological responses rather than localized brain processes. Future studies combining time-resolved metabolomics with neuroimaging or electrophysiological recordings may help disentangle these relationships and determine whether specific metabolic patterns reliably track cognitive states (Graham et al., 2015; Kaddurah-Daouk and Krishnan, 2009).

### 6.1 Integration with neuroimaging

Neuroimaging methods such as functional magnetic resonance imaging (fMRI) have transformed cognitive neuroscience by enabling the mapping of brain activity associated with specific mental processes (Logothetis, 2008; Raichle, 2015). However, fMRI primarily measures hemodynamic responses rather than the molecular mechanisms underlying neuronal signaling. MS-based metabolomics can complement neuroimaging by identifying biochemical signatures associated with neural activity and metabolic demand. Imaging mass spectrometry techniques provide an additional level of integration. Methods such as matrix-assisted laser desorption ionization imaging mass spectrometry (MALDI-IMS) enable spatial mapping of metabolites and lipids directly within brain tissue (Aebersold and Mann, 2016). When combined with anatomical or functional imaging data, these molecular maps can reveal relationships between biochemical gradients and brain structure.

### 6.2 Integration with electrophysiological techniques

Electroencephalography (EEG) and magnetoencephalography (MEG) measure electrical and magnetic signals generated by neuronal populations with high temporal resolution. These techniques are widely used to study rapid cognitive processes such as perception, attention, and decision making, but they provide limited information about the biochemical processes accompanying neural activity.

MS-based measurements can help bridge this gap by characterizing metabolic changes associated with neural oscillations and cognitive workload. Breath analysis of volatile organic compounds (VOCs), for example, can be performed during cognitive tasks using proton-transfer-reaction time-of-flight mass spectrometry (PTR-TOF-MS). Because breath sampling is non-invasive and can be repeated continuously, it is well suited for experiments involving human participants. Changes in breath metabolites such as isoprene or acetone have been linked to physiological stress responses and energy metabolism (Amann and Smith, 2013; de Lacy Costello et al., 2014). Combining electrophysiological recordings with breathomic measurements therefore offers a potential strategy for examining how metabolic processes relate to neural dynamics during cognitive tasks.

### 6.3 Integration with neurochemical sampling

In animal models and certain clinical contexts, invasive techniques allow direct sampling of neurochemicals within specific brain regions (Ungerstedt, 1991). Microdialysis enables continuous collection of extracellular fluid, which can then be analyzed using liquid chromatography-mass spectrometry (LC–MS; Kennedy et al., 2002). This approach allows sensitive detection of neurotransmitters and metabolic intermediates.

Coupling microdialysis with MS enables researchers to examine how neurochemical changes correspond to neural activity and behavior. For example, fluctuations in dopamine, glutamate, or serotonin can be monitored during learning or reward-related tasks, providing a molecular perspective on neural signaling (Schultz et al., 1997; Wise, 2004).

### 6.4 Integration with behavioral and physiological measures

Cognitive neuroscience experiments often incorporate behavioral metrics such as reaction times, accuracy, and decision outcomes, as well as physiological indicators including heart rate or skin conductance (Cacioppo et al., 2007). MS-based metabolomic or breathomic measurements can provide an additional physiological layer linking behavior with biochemical processes. Metabolic markers related to oxidative stress, lipid metabolism, or energy utilization may vary with task difficulty or emotional stimulation (McEwen, 2007). Breath VOC analysis during behavioral experiments may therefore reveal metabolic correlates of cognitive effort or emotional arousal. Because VOC measurements can be obtained non-invasively and in real time, they are particularly compatible with human cognitive experiments.

### 6.5 Integration with microbiome research

Another emerging area involves interactions between metabolic processes, the gut microbiome, and brain function. Microbial communities produce numerous metabolites and volatile compounds that can influence neural signaling through the gut–brain axis (Cryan et al., 2019). MS-based metabolomics enables detection of these compounds in biological samples. Combining metabolomic measurements with microbial sequencing can help clarify how microbial metabolism influences physiological pathways relevant to cognition and behavior.

### 6.6 Computational integration of multimodal data

Integrating metabolomic data with neuroimaging, electrophysiological, and behavioral datasets generates complex multimodal information that requires advanced computational approaches (Table 3). Machine learning and network-based analyses are increasingly used to identify patterns linking metabolic signatures with neural activity and cognitive performance (Barabási et al., 2011). These analytical frameworks may enable systems-level models that integrate molecular metabolism with large-scale brain dynamics. Several experimental strategies can be used to integrate metabolomic or breathomic measurements with established cognitive neuroscience paradigms. Examples of potential multimodal experimental designs are summarized in Table 3.

**Table 3:** Examples of potential multimodal experimental designs.

Research question	Cognitive paradigm	Neuroscience method	Metabolomic / breathomic measurement	Potential insights	References
How does cognitive load influence systemic metabolism?	Working memory tasks (e.g., n-back)	fMRI or EEG	Real-time breath VOC monitoring using PTR-TOF-MS	Identify metabolic signatures associated with increasing cognitive demand	Raichle (2015), Dienel (2019), de Lacy Costello et al. (2014)
How do emotional stressors alter metabolic responses?	Emotional stress or threat-processing paradigms	EEG, autonomic measures	Breathomics (GC–MS or PTR-MS)	Characterize metabolic correlates of stress-related neural activity	McEwen (2007), Amann and Smith (2013), Lucchi et al. (2024)
How do learning processes reshape metabolic pathways?	Learning and memory paradigms	In vivo electrophysiology or calcium imaging (animal models)	LC–MS metabolomics of brain tissue or microdialysis samples	Link metabolic pathway changes to synaptic plasticity	Harris et al. (2012), Bessières et al. (2021), Kennedy et al. (2002)
How do metabolic states affect cognitive performance?	Attention or executive function tasks	fMRI or behavioral testing	Blood metabolomics (LC–MS)	Determine how systemic metabolic states influence neural efficiency	Attwell and Laughlin (2001), Magistretti and Allaman (2015), Dienel (2019)
How does the gut–brain axis influence cognition?	Decision-making or reward paradigms	fMRI or EEG	Metabolomics combined with microbiome profiling	Identify microbiome-derived metabolites associated with cognitive processes	Cryan et al. (2019), Foster and Bray (2017), Mayer (2011)

Example experimental designs integrating metabolomic or breathomic measurements with cognitive neuroscience methods.

## 7 Methodological and conceptual challenges

**Figure 4:**
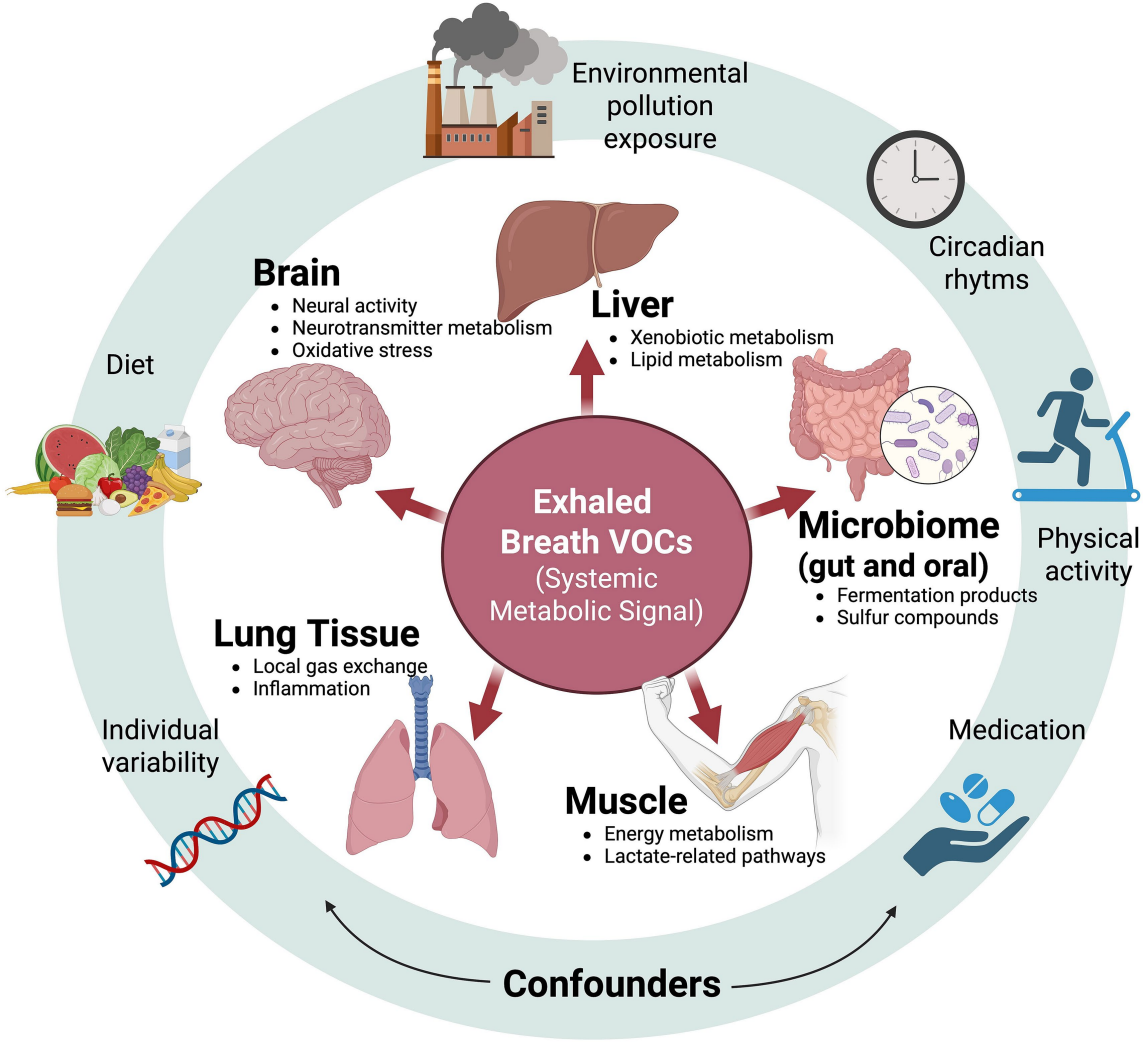
Sources of volatile organic compounds and confounding factors in breath analysis. Volatile organic compounds (VOCs) detected in exhaled breath originate from multiple biological systems, including brain metabolism, liver function, microbiome activity, muscle metabolism, and pulmonary processes. These sources contribute to systemic metabolic signals that are transported through the bloodstream and released during lung exchange. In addition, VOC profiles are influenced by external and physiological factors such as diet, environmental exposure, circadian rhythms, physical activity, medication use, and inter-individual variability. This complexity highlights a key challenge in interpreting breathomic data, as measured signals may reflect combined systemic processes rather than brain-specific metabolic activity.

Despite significant progress, several methodological and conceptual challenges remain. A central limitation concerns the biological specificity of peripheral metabolic markers (see Figure 4).

Because many metabolites detected in blood, breath, or other biofluids originate from liver metabolism, muscle activity, microbiome processes, or environmental exposure, attributing these signals directly to neural activity is challenging (Nicholson and Lindon, 2008). Addressing this issue will require multimodal experimental designs that combine metabolomic measurements with neuroimaging, electrophysiology, or computational modeling in order to establish robust links between metabolic signatures and neural dynamics.

### 7.1 Toward a metabolic framework of cognition

The evidence reviewed thus far suggests that metabolic processes are not merely supportive of neural activity, but constitute an integral component of the physiological architecture underlying cognition. Building on this premise, we propose a metabolic framework of cognition in which neural computation, cellular energetics, and systemic metabolic regulation are understood as interacting components of a unified biological system.

Within this framework, metabolism defines a set of constraints and regulatory dynamics that shape how neural processes unfold. Neuronal signaling is energetically expensive, and the availability, allocation, and efficiency of energetic resources directly influence synaptic transmission, network dynamics, and ultimately behavioral performance (Attwell and Laughlin, 2001; Harris et al., 2012; Magistretti and Allaman, 2015). Cognitive processes should therefore be understood not only in terms of information processing, but also in terms of the metabolic conditions that enable and limit such processing.

A central implication of this perspective is that metabolic signals, measurable through metabolomic and breathomic approaches, reflect distributed physiological responses that accompany neural activity. These signals do not provide direct readouts of localized brain processes; rather, they index system-level metabolic dynamics emerging from interactions between the brain, peripheral organs, and regulatory systems. From this viewpoint, metabolites and volatile organic compounds (VOCs) can be conceptualized as systemic markers of the energetic and biochemical processes associated with cognitive function.

**Figure 5:**
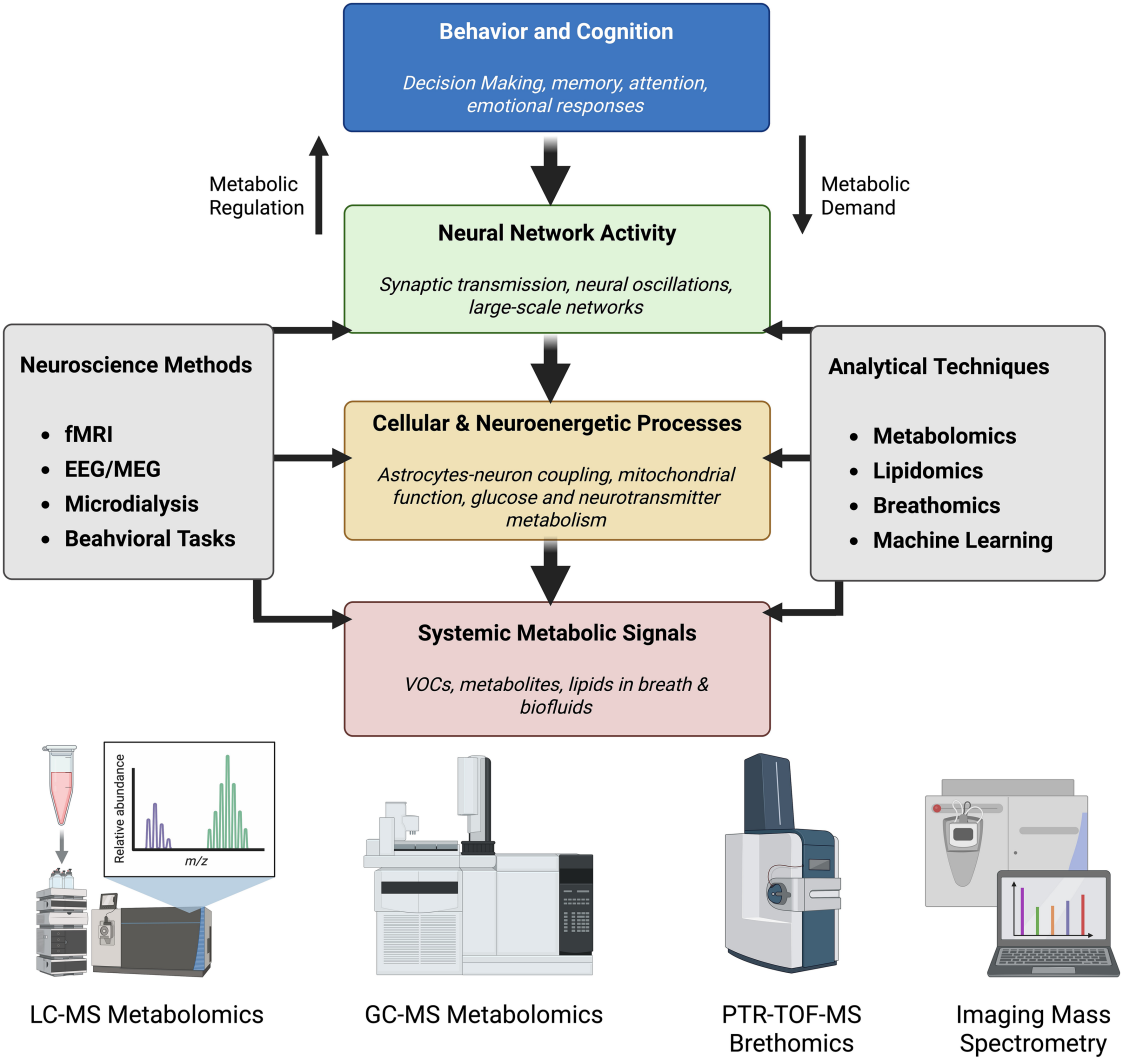
Conceptual framework linking cognition, neural activity, and metabolism. Neural activity underlying cognition generates metabolic demands that produce systemic molecular signals detectable in biofluids and breath. Integrating neuroscience methods with metabolomic and breathomic approaches provides a framework for investigating this metabolic layer connecting brain function and behavior.

Importantly, the metabolic framework extends beyond traditional neuroenergetic accounts by explicitly incorporating peripheral and whole-body physiology. While neuroenergetics has established the tight coupling between neuronal activity and cellular metabolism, the present framework emphasizes that cognitive processes are embedded within broader metabolic systems involving vascular regulation, endocrine signaling, immune responses, and microbiome activity (Cryan et al., 2019). As such, cognition emerges from coordinated interactions across multiple physiological scales rather than from brain activity in isolation. This conceptual organization of the metabolic layer linking neural activity, systemic physiology, and behavior is illustrated in Figure 5.

This perspective generates a set of testable predictions that can guide future research. First, cognitively demanding tasks should be associated with coordinated changes across neural activity and systemic metabolic markers reflecting increased energetic demand. Critically, these changes are expected to exhibit temporal structure, with metabolic responses potentially lagging behind rapid neural dynamics while still covarying with task demands. Second, experimentally manipulating metabolic states—such as through fasting, fatigue, or altered mitochondrial function—should systematically modulate cognitive performance by constraining available energetic resources. Third, multimodal experimental designs integrating neuroimaging or electrophysiology with metabolomic or breathomic measurements should reveal coupled dynamics between neural activity patterns and metabolic signatures, allowing the identification of metabolic correlates of specific cognitive states.

At the same time, the framework highlights important limitations regarding the specificity of molecular measurements. Because many metabolites and VOCs originate from multiple tissues and biochemical pathways, peripheral signals are unlikely to map uniquely onto discrete neural computations. Instead, metabolic markers are better interpreted as indicators of global or semi-specific physiological states, such as cognitive effort, stress, or metabolic activation. Disentangling these overlapping influences will require carefully controlled experimental paradigms and computational approaches capable of integrating multimodal data.

Methodologically, advancing this framework will depend on combining mass spectrometry-based measurements with established tools in cognitive neuroscience. Time-resolved metabolomics and real-time breath analysis, when paired with neuroimaging, electrophysiological recordings, and behavioral measures, offer a promising strategy for linking molecular dynamics with neural and cognitive processes. In parallel, computational approaches such as machine learning and network-based modeling may enable the identification of patterns across heterogeneous datasets, facilitating systems-level accounts of cognition (Barabási et al., 2011).

In summary, the metabolic framework proposed here shifts the focus of cognitive neuroscience from a predominantly brain-centric perspective toward an integrated view in which cognition emerges from interactions between neural activity and distributed metabolic processes. By incorporating metabolic dynamics into models of brain function, this approach provides a conceptual foundation for understanding how energetic and biochemical constraints shape cognition across brain and body systems.

## 8 Future directions

Future research integrating metabolomics, breathomics, neuroimaging, and computational modeling may enable a systems-level understanding of cognition that links neural activity with metabolic dynamics across the brain and body. Advances in portable mass spectrometry and real-time metabolomic monitoring may facilitate experiments examining metabolic responses during cognitive tasks or emotional states in naturalistic environments. Taken together, these developments suggest that metabolomic approaches may play an increasingly important role in future cognitive neuroscience research.

## 9 Conclusion

Cognitive processes are traditionally investigated through measurements of neural activity. However, the biological mechanisms that sustain neural computation extend beyond electrical signaling and hemodynamic responses. The framework proposed in this review emphasizes that cognition is embedded within a broader metabolic system linking neuronal activity with cellular energetics and whole-body physiology. By integrating metabolomic and breathomic approaches with established neuroimaging, electrophysiological, and behavioral methods, researchers may gain access to an intermediate metabolic layer connecting brain function and behavior. Although significant methodological challenges remain, particularly in distinguishing brain-derived metabolic signals from systemic physiological processes, advances in mass spectrometry, multimodal experimental design, and computational modeling are rapidly expanding the feasibility of such investigations. Incorporating metabolic dynamics into systems-level models of brain function may therefore open new avenues for understanding how cognitive processes emerge from interactions between neural circuits, cellular metabolism, and organism-level physiology.
